# Phase II study of nedaplatin and irinotecan with concurrent thoracic radiotherapy in patients with locally advanced non-small-cell lung cancer

**DOI:** 10.1038/sj.bjc.6605875

**Published:** 2010-10-12

**Authors:** F Oshita, M Ohe, T Honda, S Murakami, T Kondo, H Saito, K Noda, K Yamashita, Y Nakayama, K Yamada

**Affiliations:** 1 Department of Thoracic Oncology, Kanagawa Cancer Center, Nakao 1-1-2, Asahi-ku, Yokohama 241-0815, Japan; 2 Department of Radiotherapy, Kanagawa Cancer Center, Nakao 1-1-2, Asahi-ku, Yokohama 241-0815, Japan

**Keywords:** lung cancer, non-small cell, nedaplatin, irinotecan, radiation

## Abstract

**Background:**

Current international guidelines recommend the use of platinum-based chemotherapy with thoracic radiotherapy (TRT) for patients with locally advanced non-small-cell lung cancer (NSCLC).

**Methods:**

Patients with unresectable stage IIIA or IIIB NSCLC were treated with nedaplatin (NP) at 50 mg m^−2^ and irinotecan (CPT) at 60 mg m^−2^ on days 1 and 8 every 4 weeks for two to four cycles with concurrent TRT (2 Gy per day, total 60 Gy).

**Results:**

All 35 patients were able to receive a total of 60 Gy. Adverse effects and events in chemotherapy with TRT were grade 3 or 4 anaemia, neutropenia and thrombocytopenia, which occurred in 3.0%, 32.8% and 6.0% of patients, respectively. There was no grade 3 pneumonitis or oesophagitis. Adverse effects and events in chemotherapy alone were mild. There was no treatment-related death. An overall response rate was 94.3%. The median progression-free and overall survivals were 13.0 and 36.0 months, respectively. The 5-year disease-free and overall survival rates were 25.7% and 40.0%, respectively.

**Conclusion:**

NP and CPT treatment with concurrent TRT is effective and safe for patients with unresectable, locally advanced NSCLC.

Locally advanced stage III non-small-cell lung cancer (NSCLC) can be thought of as a two-compartment model: a local-regional compartment in the chest and a distant compartment harbouring potential micrometastases. At the most basic level, thoracic radiation therapy (TRT) is directed towards the intrathoracic tumour burden, whereas chemotherapy works to eradicate systemic microscopic metastatic deposits below current levels of detection by computerised tomography scanning or positron emission imaging with fluorodeoxyglucose. Chemotherapy may contribute a radiosensitising effect locally, as well as providing cytoreduction of bulky locoregional disease. Four different treatment paradigms have emerged in recent years for application of chemoradiotherapy (CRT): sequential, concurrent, induction chemotherapy followed by concurrent CRT and concurrent CRT followed by consolidation chemotherapy (Gandara *et al*, 2005). Current international guidelines recommend the use of platinum-based chemotherapy with TRT for patients with locally advanced NSCLC (Pfister *et al*, 2004). A randomised phase III study comparing induction chemotherapy with concurrent CRT using an identical chemotherapy regimen demonstrated that chemotherapy using cisplatin, vindesine and mitomycin C with concurrent TRT significantly improved survival in comparison with a sequential approach such as chemotherapy followed by TRT (Furuse *et al*, 1999). The study demonstrated that median survival time (MST) and 5-year survival rate were 16.5 months and 16% in the concurrent arm compared with 13.3 months and 9%, respectively. However, increased toxicity was noted with concurrent therapy, primarily consisting of intensified toxicities within the TRT field, notably oesophagitis with associated nutritional problems and potential dehydration. There may also be an increased risk of pneumonitis and severe myelotoxicities. Thus, concurrent TRT may be too toxic for selected patient groups with NSCLC, especially for elderly patients and those with poor performance status (PS).

Nedaplatin (NP) is an analogue of cisplatin, showing relatively low neurotoxicity and nephrotoxicity, and high *in vivo* bioavailability, ensuring the position of NP as a primary chemotherapeutic agent for the treatment of patients with advanced lung cancer (Kameyama *et al*, 1990). Our previous phase I/II study of NP and irinotecan (CPT) showed high activity against NSCLC, including a 31.0% response rate (RR), an MST of 341 days and a 1-year survival rate of 45.2% (Oshita *et al*, 2003). Mild toxicities were also demonstrated, and a subsequent phase II study of this combination demonstrated its efficacy and feasibility for elderly patients with NSCLC (Oshita *et al*, 2004). Three-dimensional analysis models have demonstrated a remarkable synergistic interaction of concurrent NP with CPT (Kanzawa *et al*, 2001), and we expected that infusion of the two drugs on the same day combined with concurrent TRT would yield a stronger effect. Some patients with locally advanced unresectable NSCLC have received sequential TRT after completion of NP and CPT chemotherapy at the Kanagawa Cancer Center. These patients experienced only mild localised lung damage in the radiation field after completion of full-dose TRT. These data suggested that chemotherapy using NP and CPT would be feasible when combined with concurrent TRT. Therefore, we conducted a phase II study to examine the feasibility and effect of NP and CPT concurrent with TRT, planning to combine TRT with the first course of NP and CPT chemotherapy.

## Patients and methods

The institutional review board of the Kanagawa Cancer Center reviewed and approved this study before commencement.

### Patients

Patients with histologically or cytologically confirmed NSCLC were registered. Eligibility criteria were clinical stage IIIA or IIIB, cytologically proven N2, unresectable cancer, an expected survival of at least 12 weeks, a TRT field less than half of the unilateral lung, patient age <70 years, Eastern Cooperative Oncology Group PS score ⩽1, leukocyte count ⩾4000 per *μ*l, haemoglobin ⩾10 g per 100 ml, platelet count ⩾100 000 per *μ*l, total serum bilirubin ⩽1.5 mg per 100 ml, aspartate aminotransferase and alanine aminotransferase ⩽90 IU l^−1^, serum creatinine ⩽1.5 mg per 100 ml and PaO_2_ ⩾70 torr. None of the patients had received chemotherapy, radiotherapy or surgical resection previously. Patients with pleural or pericardial effusion were excluded. Written informed consent was obtained from every patient.

### Chemotherapy and TRT

Nedaplatin was administered at a dose of 50 mg m^−2^ on days 1 and 8. Irinotecan was also administered at a dose of 60 mg m^−2^ on days 1 and 8. Patients were given a 5-HT_3_ antagonist and dexamethasone intravenously before administration of the anticancer drugs on days 1 and 8. Subsequent cycles of chemotherapy were started when the patients satisfied the organ function criteria: leukocyte count ⩾3000 per *μ*l, neutrophil count ⩾1500 per *μ*l, platelet count ⩾75 000 per *μ*l and less than grade 1 non-haematological toxicities, except alopecia. If the dose-limiting toxicity (DLT) was reached, the dose of NP and CPT in the subsequent cycle was reduced by 10 mg m^−2^. Dose reduction was allowed once, and any patient who experienced DLT twice was withdrawn from the protocol. Dose-limiting toxicity was defined as toxicity in every cycle consisting of grade 4 neutropenia lasting 4 days or more; grade 4 neutropenia with fever of 38°C or higher; grade 4 thrombocytopenia; ⩾grade 2 depression of PaO_2_; ⩾grade 2 dyspnoea; or grade 3 or 4 other non-haematological toxicity, except alopecia, nausea and vomiting. Physical examination, a complete blood cell count, biochemical tests and chest radiography were performed weekly. Chemotherapy was repeated for a maximum of four cycles, unless the disease progressed, but was stopped if the tumour response was judged to be stable disease (SD) after two cycles.

Thoracic radiotherapy using photon beams from a linac or microtron accelerator, with energy between 6 and 10 MV at a single dose of 2 Gy once daily, 5 days per week, total 60 Gy, was begun on day 1 or 2 of the first cycle of NP and CPT chemotherapy. The clinical target volume was based on conventional chest X-ray and CT scans, and included the primary lesion (CTV1), involved lymph nodes with a short diameter of 1 cm or larger (CTV2) and the ipsilateral pulmonary hilum and bilateral mediastinum area (CTV3). Anterior and posterior parallel opposed fields encompassed the initial planned target volume (PTV), consisting of CTV1–3, with the superior and inferior field margins extended to 1.5 cm and lateral field margins extended to 1.5 cm to allow for respiratory variation and fixation error. The spinal cord dose was limited to 50 Gy using oblique parallel opposed fields. When grade 4 leukopenia, neutropenia or thrombocytopenia, fever ⩾38°C, grade 2 pneumonitis or other grade 3 or 4 non-haematological toxicities appeared, radiation therapy was stopped until the toxicities ameliorated.

### Evaluation of response and toxicities

Tumour response was evaluated according to the RECIST criteria (Therasse *et al*, 2000). Complete response (CR) was defined as the complete disappearance of all evidence of tumour for at least 4 weeks. Partial response (PR) was defined as at least a 50% reduction in the sum of the product of the two greatest perpendicular diameters of all indicator lesions or a reduction of more than 50% in evaluable disease for at least 4 weeks, with no appearance of new lesions or progression of any existing lesions. Progressive disease (PD) was defined as at least a 25% increase in the tumour area or the appearance of new lesions. All other outcomes were classified as SD. Toxicities were evaluated according to the National Cancer Institute-Common Toxicity Criteria ver. 2 criteria (http://ctep.cancer.gov/protocolDevelopment/electronic_applications/docs/ctcv20_4-30-992.pdf).

### Study design

We chose an 80% RR as a desirable target level and a 60% RR as uninteresting. The study design had power in excess of 95% and <20% error, and therefore 13 assessable patients in the first step and 22 in the second step were required according to the minimax design of Simon (1989). We decided to stop the study if there were fewer than nine responders in the first step. The regimen was defined as active if there were 26 or more responders out of the total of 35 patients. Overall, survival was estimated by the method of Kaplan and Meier.

## Results

### Patient characteristics

Between August 2002 and June 2005, 35 patients were registered in the phase II study (Table 1). A total of 22 patients were registered for assessment of response in the first stage. Of the 22 patients in the first stage, 20 responded and 13 patients were registered in the second stage. A total of 25 patients were male and 10 were female, with a median age of 62 years (range 43–69 years). Nine patients had a PS of 0 and 26 had a PS of 1. In all, 22 patients had adenocarcinoma, 10 had squamous cell carcinoma and the remaining patients had other cancers. A total of 30 patients had clinical stage IIIA and five had stage IIIB. Every patient was proven cytologically to have N2 disease by broncoscopic examination.

### Treatment delivery

A total of 67 cycles were administered with concurrent TRT. After completion of TRT, a total of 48 cycles were administered. A total of 28 patients received three or four cycles of chemotherapy, and the median number of chemotherapy cycles was four. Chemotherapy dose reduction was required in two patients in the second cycle and in one patient in the fourth cycle because of grade 4 neutropenia lasting 4 days or more. Three patients were unable to continue the second cycle of chemotherapy because of grade 2 pneumonitis in two and delayed neutropenia in one. The reasons for discontinuing chemotherapy after two cycles were PD (bone metastasis) in two cases and patient refusal in two cases. All 35 patients were able to receive a total of 60 Gy of TRT.

### Toxicities

Adverse effects and events in cycles 1 and 2 of chemotherapy with concurrent TRT are summarised in Table 2. Grade 3 or 4 anaemia, leukopenia, neutropenia and thrombocytopenia occurred in 3.0, 38.8%, 32.8% and 6.0%, respectively. There were no grade 4 toxicities, except for leukopenia and neutropenia. There was no grade 3 pneumonitis or oesophagitis. Adverse effects and events in cycles 3 and 4 of chemotherapy are summarised in Table 3. Grade 3 or 4 anaemia, leukopenia, neutropenia and thrombocytopenia occurred in 10.4%, 35.4%, 41.7% and 16.7%, respectively. There were no grade 3 or 4 non-haematological toxicities, or any case of treatment-related death.

### Response and survivals

**Figure 1:**
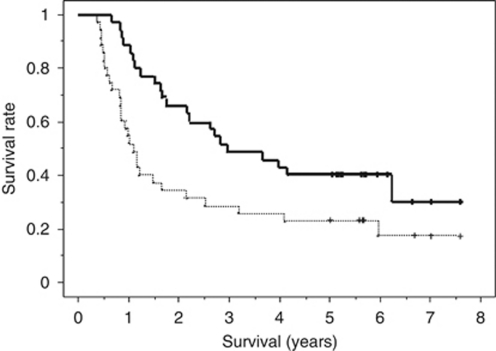
Progression-free (a dotted line) and overall (a bold line) survival curves constructed using the Kaplan–Meier method. The median progression-free survival was 13.0 months (range 4.6 to 91.0+ months) and 5-year disease-free survival rate was 25.7%. The MST was 36.0 months (range 8.0 to 91.0+ months) and 5-year survival rates were 40.0%.

One patient achieved CR, 32 achieved PR and 2 showed SD, and the overall RR in this series was 94.3% (95% confidence interval 86.6–100%). The median progression-free survival was 13.0 months (range 4.6 to 91.0+ months) and 5-year disease-free survival rate was 25.7% (Figure 1). The MST was 36.0 months (range 8.0 to 91.0+ months) and the 1-, 3- and 5-year survival rates were 88.6%, 51.4% and 40.0%, respectively (Figure 1).

### Sites of first failure and second treatments

In all, 26 patients had recurrences during follow-up. The first failure was observed at the primary site in the radiation field in nine patients, and distant failure was detected in 17 patients (Table 4). Five of six patients, in whom the site of first failure was the brain, received whole-brain irradiation. In all 15 patients received second-line chemotherapies, such as docetaxel-based chemotherapy in 6, gefitinib (Gef) in 6 and others in 3. Two patients received radiotherapy to metastatic sites and five received supportive care only. Four patients had second primary malignancies: two in the oesophagus, one in the kidney and one in the lung. Three of them received surgical resections and are alive and disease free.

## Discussion

Clinical trials and a meta-analysis have shown that concurrent chemotherapy and TRT affords outcomes superior to those of sequential therapy (Furuse *et al*, 1999; Zatloukal *et al*, 2004; Fournel *et al*, 2005). As the cisplatin and etoposide combination can be given at full dose, concurrent with TRT, this doublet is considered as the standard option for CRT protocols, as shown in two sequential, non-randomised studies by the Southwest Oncology Group (SWOG) (Albain *et al*, 2002; Gandara *et al*, 2003). However, there have been no phase III comparisons of different CRT regimens. Therefore, a specific standard regimen cannot be established at this time.

The present regimen comprising NP and CPT with concurrent TRT showed extremely high activity. Both the MST and 5-year survival rate were the best recorded so far for a phase II study in patients with unresectable locally advanced NSCLC. Only four patients died within 1 year, and nine patients, accounting for a quarter of the total subjects, survived for over 5 years without tumour recurrence. We anticipated the toxicities that would be induced by this combined modality, and administered the NP in divided doses on days 1 and 8, unlike previous studies (Oshita *et al*, 2003, 2004). As three-dimensional analysis models have demonstrated that concurrent exposure to NP and CPT produces a marked synergistic interaction (Kanzawa *et al*, 2001), it was considered that a more potent effect would be achieved by infusing the two drugs on the same day.

The present regimen comprising NP and CPT with concurrent TRT also demonstrated high feasibility. Haematological toxicities were mild, and oesophagitis and pneumonitis were manageable. Grade 3/4 neutropenia occurred only in 32.8% of patients, and grade 3 febrile neutropenia occurred in only 9.0% during the concurrent phase with TRT. Every febrile episode was improved by antibiotics, and every patient was able to receive a subsequent cycle of chemotherapy. Other than febrile neutropenia, grade 3 non-haematological toxicities included only one episode each of fatigue and vomiting, and there was no grade 3/4 pneumonitis or oesophagitis. Nine patients experienced grade 1/2 pneumonitis in the concurrent phase of CRT, and two of them were unable to progress to the second cycle of NP and CPT chemotherapy, although they did receive the full dose of TRT. Pneumonitis induced by combination therapy with TRT and CPT was anticipated in some studies. Wu and Choy (2002) reviewed some trials and recommended CPT as a radiosensitiser, but gave a warning of occurrence of pneumonitis. Full dose of cisplatin and CPT with concurrent TRT resulted in 10% of patients with grade 3 pneumonitis in a Japanese trial (Fukuda *et al*, 2007) and concurrent TRT with carboplatin and CPT resulted in a 42% incidence of grade 2 or more radiation pneumonitis in a recent American study (Bastos *et al*, 2010). However, pneumonitis in this study was grade 2 or less and only one late grade 3 pneumonitis occurred in a study of weekly CPT and cisplatin treatment with concurrent TRT (Langer *et al*, 2007). These data suggested that TRT with full dose of platinum and CPT might induce some amount of severe radiation pneumonitis but that with weekly or divided platinum and CPT, as in our study, might be able to decrease the grade of pneumonitis. There was no treatment-related death, and the shortest survival time was 8 months. A Japanese trial of docetaxel consolidation therapy after cisplatin, vinorelbine and concurrent TRT in patients with unresectable stage III NSCLC reported grade 3/4 neutropenia, oesophagitis and pneumonitis in 63.9%, 11.3% and 3.1% of patients, respectively, in the phase of cisplatin and vinorelbine with concurrent TRT (Sekine *et al*, 2006). Another recent Japanese trial of cisplatin and S-1 with concurrent TRT in patients with unresectable stage III NSCLC reported grade 3/4 neutropenia, grade 3 oesophagitis and grade 3 pneumonitis in 32.0%, 10.0% and 5.0% of patients, respectively, in the cisplatin and S-1 with concurrent TRT phase (Ohyanagi *et al*, 2009). The SWOG 9504 also demonstrated moderate toxicities, such as grade 3 or 4 neutropenia, oesophagitis and pneumonitis developing in 74%, 17% and 5%, respectively, in the cisplatin and etoposide with concurrent TRT phase (Gandara *et al*, 2003). These three trials demonstrated MSTs of 26, 30 and 33.1 months, which are comparable with the results of the present study. Thus, the present combined modality yielded favourable outcomes with low toxicities. This powerful NP and CPT combination chemotherapy also demonstrated high safety in lung cancer patients, with multiple risk factors in a retrospective analysis (Oshita *et al*, 2007). A total of 31 NSCLC patients with multiple high-risk factors were treated with NP at 50 mg m^−2^ and with CPT at 50 mg m^−2^ on days 1 and 8 every 4 weeks. With regard to toxicities, 7 (8.4%) and 11 cycles (13%) were associated with grade 4 neutropenia and grade 3 febrile neutropenia, respectively. Each of the toxicities was controllable, and there was no treatment-related death. One patient achieved CR, 13 achieved PR and the overall RR was 45.2%.

Nedaplatin and CPT with concurrent TRT yielded a high RR and good outcome, but 26 of the 35 treated patients had tumour recurrence. Recurrent lesions were located in both the primary irradiated field of the lung and in distant organs such as the brain or bones. Tumour regrowth within the radiated field and brain metastasis were detected as the first sites of recurrence in nine and six patients, respectively. Thus, the present treatment, although powerful, seems to be somewhat insufficient for both locoregional control and suppression of distant metastasis. Brain metastasis as the first site of relapse was observed in six patients within 1 year. To suppress brain metastasis, prophylactic cranial irradiation after completion of CRT could be considered. This approach has been established in the treatment of small-cell lung cancer, but has not been recommended for stage III NSCLC. As only 23.1% of our study patients demonstrated a single brain metastasis, prophylactic radiotherapy should be considered carefully.

The use of an additional chemotherapy component remains investigational. The consolidation chemotherapy approach and the addition of targeted therapies to concurrent TRT are currently under investigation. Careful consideration must be given to the exact role a novel therapeutic agent is expected to have in a combined modality therapy setting; that is, in addition to CRT, should the agent be a single drug active against NSCLC, a radiosensitising agent or an agent to control distant micrometastasis? Additional data regarding docetaxel consolidation have been obtained from the SWOG and also from trials in Japan. A non-randomised study by the SWOG evaluated docetaxel consolidation after cisplatin and etoposide CRT, and suggested that 75 mg m^−2^ docetaxel was better tolerated without loss of efficacy (Gandara *et al*, 2003). In a phase II trial in Japan, 97 patients with unresectable stage III NSCLC received docetaxel consolidation after concurrent CRT using cisplatin and vinorelbine for three cycles (Sekine *et al*, 2006). Only 37% of patients completed all three cycles of docetaxel. Pneumonitis was the most common reason for early discontinuation, and four patients died of this complication.

Tyrosine kinase inhibitors directed against epidermal growth factor receptor (EGFR) have been shown to be effective for the treatment of advanced NSCLC (Fukuoka *et al*, 2003). A retrospective study has demonstrated that NSCLC patients with EGFR mutation have a better outcome with Gef treatment than do patients with wild-type EGFR (Mitsudomi *et al*, 2005). A randomised phase III study comparing Gef with standard carboplatin plus paclitaxel has demonstrated that Gef conferred significantly superior progression-free survival as first-line chemotherapy in patients with EGFR mutation (Mok *et al*, 2009). Furthermore, another phase III trial demonstrated that chemotherapy-naïve patients with EGFR have longer progression-free survival if they were treated with Gef than if they were treated with cisplatin and docetaxel (Mitsudomi *et al*, 2010). These data suggested that Gef is essential for NSCLC patients with EGFR mutation. With regard to the use of EGFR tyrosine kinase inhibitors given concurrently with TRT or CRT, the Cancer and Leukemia Group B30106 is a stratified phase II trial testing Gef concurrently with TRT alone or with CRT in patients with NSCLC (Ready *et al*, 2004), but the feasibility of Gef combined with CRT or TRT has not been established. Our recent study demonstrated that sequential Gef treatment added to NP and CPT combination chemotherapy conferred a survival benefit (32.1%, 2-year survival rate) in 28 elderly patients with advanced NSCLC, whose EGFR mutation was unknown (Oshita *et al*, 2008). Thus, it was suggested that CRT followed by Gef maintenance strategy should be explored for locally advanced NSCLC with EGFR mutation.

In conclusion, NP and CPT with concurrent TRT have been shown to have high activity with high safety. A phase III study will be required to establish this regimen as a new standard therapy against unresectable locally advanced NSCLC.

## Figures and Tables

**Table 1:** Patient characteristics

	**No. of patients**
Total	35
	
*Age* (*years*)	
Median	62
Range	(43–69)
	
*Gender*	
Male	25
Female	10
	
*PS* (*ECOG*)	
0	9
1	26
	
*Pathology*	
Adenocarcinoma	22
Squamous cell carcinoma	10
Others	3
	
*Clinical stage*	
IIIA	30
IIIB	5

Abbreviation: ECOG=Eastern Cooperative Oncology Group.

**Table 2:** Adverse effects and events in chemotherapy and concurrent thoracic radiation

	**Grade (NCI-CTC ver.2)**					
**Toxicity**	**0**	**1**	**2**	**3**	**4**	**Percentage of G3 and 4**
Haemoglobin	9	46	10	2	0	3.0
Leukocytes	5	7	29	25	1	38.8
Neutrophils	8	14	23	18	4	32.8
Platelets	32	27	4	4	0	6.0
Bilirubin	57	8	2	0	0	—
Creatinine	67	0	0	0	0	—
SGOT	55	11	1	0	0	—
SGPT	49	16	2	0	0	—
Fatigue	0	60	6	1	0	1.5
Nausea/vomiting	46	17	3	1	0	1.5
Diarrhoea	44	23	0	0	0	—
Pneumonitis	58	5	4	0	0	—
Fever	59	7	1	0	0	—
Febrile neutropenia	61	—	—	6	0	9.0
Neuropathy–sensory	64	3	0	0	0	—
Alopecia	44	23	0	0	0	—
Oesophagitis	36	28	3	0	0	—
Gastritis	62	5	0	0	0	—
Cystitis	66	1	0	0	0	—
Sense of smell/dysgeusia	59	8	0	0	0	—
Constipation	61	6	0	0	0	—
Stomatitis	64	3	0	0	0	—
Epistaxis	66	1	0	0	0	—

Abbreviations: NCI-CTC ver.2=National Cancer Institute-Common Toxicity Criteria; SGOT=serum glutamic oxaloacetic transaminase; SGPT=Serum glutamic pyruvic transaminase.

**Table 3:** Adverse effects and events in 3 and 4 courses of chemotherapy

	**Grade (NCI-CTC ver.2)**					
**Toxicity**	**0**	**1**	**2**	**3**	**4**	**Percentage of G3 and 4**
Haemoglobin	4	27	12	5	0	10.4
Leukocytes	10	7	14	15	2	35.4
Neutrophils	13	4	11	12	8	41.7
Platelets	24	10	6	7	1	16.7
Bilirubin	45	2	1	0	0	—
Creatinine	47	1	0	0	0	—
SGOT	42	6	0	0	0	—
SGPT	40	8	0	0	0	—
Fatigue	0	45	3	0	0	—
Nausea/vomiting	38	10	0	0	0	—
Diarrhoea	35	13	0	0	0	—
Pneumonitis	35	9	4	0	0	—
Fever	47	1	0	0	0	—
Febrile neutropenia	46	—	—	2	0	4.0
Neuropathy–sensory	48	0	0	0	0	—
Alopecia	17	31	0	0	0	—
Oesophagitis	44	4	0	0	0	—
Gastritis	45	3	0	0	0	—
Cystitis	48	0	0	0	0	—
Sense of smell/dysgeusia	43	5	0	0	0	—
Constipation	46	2	0	0	0	—
Stomatitis	47	1	0	0	0	—

Abbreviations: NCI-CTC ver.2=National Cancer Institute-Common Toxicity Criteria; SGOT=serum glutamic oxaloacetic transaminase; SGPT=Serum glutamic pyruvic transaminase.

**Table 4:** Sites of initial failure

	**Patients**	
**First site of failure**	**No.**	**Percentage**
Local	9	34.6
		
*Distant*		
Brain	6	23.1
Bone	4^a^	15.4
Lung	4^a^	15.4
Cervix	3	11.5
Liver	1	3.8
Kidney	1	3.8
Adrenal	1	3.8

a Three patients had recurrence in both bone and lung.
